# Long-Term Sickness Absence Due to Mental Disorders Is Associated with Individual Features and Psychosocial Work Conditions

**DOI:** 10.1371/journal.pone.0115885

**Published:** 2014-12-22

**Authors:** João Silvestre da Silva-Junior, Frida Marina Fischer

**Affiliations:** 1 National Social Security Institute, Ministry of Social Security, São Paulo, SP, Brazil; 2 Department of Environmental Health, School of Public Health, University of São Paulo, São Paulo, SP, Brazil; University of Pennsylvania, United States of America

## Abstract

**Aims:**

Sickness absence is a socioeconomic global burden. In Brazil, mental disorders are the third leading cause of social security benefits payments. The aim of the present study was to compare factors associated with long-term sickness absence between workers who claimed social benefits due to mental disorders or by other causes. We investigated individual features and occupational characteristics. In addition, we evaluated psychosocial factors at work assessed by the Demand-Control-Support (DCS) and Effort-Reward Imbalance (ERI) models, and whether they were associated with long-term sickness absence due to mental disorders (LTSA-MD).

**Methods:**

The present case-control study was conducted in São Paulo, Brazil. The sample (n = 385) included workers on sick leave for more than 15 days. Cases were the participants with disabling psychiatric illnesses, and controls were the ones with other disabling diseases. Interviews were conducted to assess individual features (sociodemographic data, health habits/lifestyle, health conditions) and occupational characteristics. The participants' perception of exposure to dimensions of the DCS and ERI models was also recorded. Multiple logistic regressions were performed to evaluate the association between independent variables and LTSA-MD.

**Results:**

All the regression analyses showed that LTSA-MD was associated with female sex, self-reported white skin color, higher education level, high tobacco consumption, high alcohol intake, two or more comorbidities, exposure to violence at work, high job strain and low social support at work, effort-reward imbalance and high overcommitment to work. LTSA-MD was associated with separate and combined DCS and ERI stress models.

**Conclusions:**

Individual features and work conditions were associated with LTSA-MD. Combined analysis of stress models showed that psychosocial factors at work were significantly associated with LTSA-MD. Resourceful use of this information may contribute to the implementation of preventive actions and strategies to facilitate return to work targeting the populations most susceptible to mental disorders.

## Introduction

Work is a part of a process of social integration that directly affects the physical and mental health of individuals. Sickness absence is an indicator of the state of health of workers [1]. Sociodemographic characteristics [2]–[4], health habits and lifestyles [2]–[5], environmental work conditions [2], [5]–[6], psychosocial factors at work [5] and the individuals' state of health [7] are some of the factors associated with sickness absence.

Sick leave due to mental disorders is a global cause of concern that deserves attention as a function of its associated costs [8]. In Brazil, sick leaves lasting up to 15 days are paid by the worker's employer, while workers on longer leaves must claim sickness benefits from the public social security system (National Social Security Institute - INSS). Applicants are evaluated by medical experts to establish whether the sick leave benefits should be granted. In recent years, mental and behavioral disorders became the third leading cause of temporary sickness benefits. About 203,000 new sick leave benefits due to mental problems were granted from 2008 to 2011, costing more than US$ 90 million to the Brazilian public pension system [9].

An earlier joint publication by the International Labour Office and the World Health Organization (ILO/WHO) called the attention to the relevance of the psychosocial factors at work for the employees' state of health. Long-term exposure to such stressors is associated with psychosomatic complaints, psychiatric symptoms and changes in wellbeing [10]. Theoretical models were developed to define and explain the effects of occupational stress on the workers' health, two among which were given special attention, to wit, the Demand-Control-Support (DCS) [11] model, which is assessed by means of the Swedish Demand-Control-Support Questionnaire [12], and the Effort-Reward Imbalance (ERI) model [13], which is also assessed by means of a specific questionnaire [14]. According to the literature, there is overlap between those questionnaires dimensions [15]. Analyses combining both models provide better estimates of the effects of stressful experiences at the workplace [16].

Recent studies on sick leave applied the DCS and ERI models to the investigation of psychosocial factors at work, either alone or in combination [17]–[20]. However, those models have not yet been used in combination to the investigation of such factors and LTSA-MD.

A first spell of sickness absence due to mental disorders is associated with risk for recurrence of further sick leaves [21]. For that reason, an accurate understanding of the factors that are related with sickness absence might increase the efficacy of preventive actions and point to strategies to facilitate return to work [22].

The aim of the present study was to compare factors associated with long-term sickness absence between workers who claimed social benefits due to mental disorders or by other causes. We investigated individual features and occupational characteristics. In addition, we evaluated psychosocial factors at work assessed by the DCS and ERI models, and whether they were associated with the outcome variable.

## Material and Methods

The present case-control study was performed at the largest public social security branch in the city of São Paulo, Brazil, in 2011. The group of patients assigned to each medical expert was considered to represent a cluster. Every day along the study period, and at each work shift, clusters were randomly selected to compose the study population. After the completion of the social security medical evaluation, the participants from the randomly selected clusters who met the inclusion criteria were referred for interview with one of the investigators.

The participants must have worked in formal employment and requested sick leave benefits after having being off from work for more than 15 days. The ones who had dual employment or were out of work for more than 12 months were excluded.

A total of 438 eligible individuals were invited to participate in the study, being that 53 refused. Difference in sex and medical diagnosis was not found among the individuals who agreed or refused to participate, however, the former where younger than the latter (p<0.05), being that their average age was 34.8 (±9.5) and 38.0 (±9.44) years old, respectively.

The final study population comprised 385 participants. As cases were defined 160 volunteers who requested sick leave benefits after having being diagnosed with one of the “Mental and behavioral disorders” listed in International Statistical Classification of Diseases and Related Health Problems - 10^th^ Revision (ICD-10) Chapter 5. Most of the sample was characterized as having: “Mood [affective] disorders - F30-F39” (53.7%) and “Neurotic, stress-related and somatoform disorders – F40- F49” (32.6%). Other mental and behavioral disorders found were: “Organic, including symptomatic, mental disorders F00-F09) - (1.9%), “Mental and behavioral disorders due to psychoactive substance use - F10-F19” (5%), “Schizophrenia, schizotypal and delusional disorders - F20-F29” (4.9%) and “Disorders of adult personality and behavior - F60-F69” (1.9%).

Controls were 225 participants who applied for sickness benefits due to diseases listed in any other ICD10 chapter, being their distribution as follows: chapter XIX - Injury, poisoning and certain other consequences of external causes (36.9%), XIII - Diseases of the musculoskeletal system and connective tissue (19.6%), XI - Diseases of the digestive system (7.6%), IX - Diseases of the circulatory system (5.3%), II - Neoplasms (5.3%), and others (25.3%).

All the participants signed an informed consent form in compliance with the Declaration of Helsinki. The study was authorized by INSS and approved by the ethics committee of School of Public Health, University of São Paulo, Brazil.

Interviews were conducted to fill the forms used to collect data on covariates that could show association with sickness absence [2]–[7]; [17]–[20]; [23]. The investigated individual features were sociodemographic data (sex, age, self-reported skin color, marital status, education), health habits and lifestyle (tobacco use, alcohol intake, physical activity), and health conditions (comorbidities, body mass index).

The versions validated for the Brazilian Portuguese language of the following questionnaires were used to evaluate the participants' health habits and lifestyle: Fagerström Tolerance Questionnaire for tobacco use [24], Alcohol Use Disorders Identification Test (AUDIT) for level of alcohol intake [25] and International Physical Activity Questionnaire (IPAQ) [26].

Morbidities that required medical care in the year previous to data collection were investigated to establish the participants' health conditions. The volunteers' height and weight were measured at the time of the interview to calculate the body mass index (BMI).

The occupational data assessed were: nature of work and employment status, current job position and violence at the workplace. The psychosocial factors at the workplace assessed by the DCS model were investigated using the version validated for the Brazilian Portuguese language [27] of the “Swedish Demand-Control-Support Questionnaire” (DCSQ). A short version of an ERI Brazilian Portuguese questionnaire was used to assess dimensions of the ERI model [28].

The diagnosis of the sick leave-related illnesses was obtained from the reports issued by the medical experts from the INSS branch where the study was conducted. The allocation of participants reporting more than one disease to the group of cases or controls was decided based on their main diagnosis, as established by the INSS examiner.

Univariate logistic regression models were built including the outcome and the independent variables. Variables with p-value equal to or lower than 0.20 on univariate regression were selected for the stepwise forward process used to fit multiple logistic models. A correlation matrix was elaborated to assess the potential overlap effect of variables. P-value less than 0.05 was considered to be significant in the final model.

Four final regression models were built to evaluate the psychosocial factors at work and their association with LTSA-MD. Regression analysis (A) evaluated the association of LTSA-MD with the interaction of three psychosocial dimensions assessed by the DCS model. Regression analysis (B) investigated the association of the outcome with the interaction of three dimensions assessed by the ERI model. Regression analysis (C) included both models separately as covariates associated with the outcome. Finally, regression analysis (D) assessed the interaction of both models and their association with LTSA-MD.

Software Epidata version 3.1 was used for data collection. The data were tabulated and analyzed using software Epi-Info version 3.5.2 and SPSS version 19.

## Results

The study population was mostly composed of women (56.6%), with mean age of 34.8 (±9.5) years old. Most participants self-reported to be white (49.1%), live with someone (married/stable relationship) (51.4%), and had attended 11 years or more of formal education (67.7%) (Table 1).

**Table 1:** Distribution of participants according individual features, São Paulo, Brazil, 2011 (N = 385).

	Case (N = 160)	%	Mean (SD)	Control (N = 225)	%	Mean (SD)	p-value
**Sex**							
Male	48	28.7		119	71.3		
Female	112	51.4		106	48.6		<0.001
**Age**			34.21 (8.13)			35.17 (10.37)	0.325
**Self-reported skin color**							
White	92	48.7		97	51.3		
Black	46	32.4		96	67.6		
Other	22	40.7		32	59.3		0.053
**Marital status**							
Single	54	40.0		81	60.0		
Married/stable relationship	83	41.9		115	58.1		
Separated/divorced/widower	21			27			0.625
**Education (formal schooling in years)**							
<11	34	27.4		90	72.6		
> = 11	126	48.3		135	51.7		0.001
**Tobacco use**							
None	130	41.5		183	58.5		
Low	13	26.5		36	73.5		
High	17	73.9		6	26.1		0.1617
**Alcohol intake**							
None	143	45.1		174	54.9		
Low	12	19.7		49	80.3		
High	5	71.4		2	28.6		0.036
**Physical activity**							
None	36	36.0		64	64.0		
Yes	124	43.5		161	56,5		0.191
**Comorbidities**							
0 or 1	17	15.5		93	84.5		
> = 2	143	52.0		132	48.0		<0.001
**Body mass index**							
Low weight	7	58.3		5	41.7		
Normal weight	58	38.2		94	61.8		
Overweight	51	39.8		77	60.2		
Obesity	44	47.3		49	52.7		0.427

A total of 81.3% participants were nonsmokers, and 82.3% reported being abstainer. Physical activity in the previous month was reported by 74% of the participants (Table 1).

Most participants worked in the private sector (82.6%), being that “service provider” was the most prevalent professional group (61.6%) (Table 2).

**Table 2:** Distribution of participants according to occupational characteristics, São Paulo, Brazil, 2011 (N  =  385).

	Case (N = 160)	%	Control (N = 225)	%	p-value
Employment and status					
Private sector (outsourced)	19	38.0	31	62.0	
Private sector (regular)	129	40.6	189	59.4	
Public sector*	12	70.6	5	29.4	0.103
Occupational groups					
Operational-manufacturing	11	17.7	51	82.3	
Management positions**	7	33.3	14	66.7	
Technical-administrative*	30	46.2	35	53.8	
Service providers*	112	47.3	125	52.7	0.046
Violence at workplace					
None	9	12.0	66	88.0	
Yes*	151	48.7	159	51.3	<0.001
Demand-Control quadrant					
High strain*	92	53.2	81	46.8	
Low strain	7	19.4	29	80.6	
Active work**	55	37.9	90	62.1	
Passive work	6	19.4	25	80.6	<0.001
Social support					
Low*	86	69.9	37	30.1	
High	74	28.2	188	71.8	<0.001
DCS model					
No exposure	99	32.7	204	67.3	
High strain + low social support	61	74.4	21	25.6	<0.001
Effort-Reward ratio					
<1.00	64	26.8	175	73.2	
> = 1.00 *	96	65.8	50	34.2	<0.001
Overcommitment					
Low	21	15.2	117	84.8	
High*	139	56.3	108	43.7	<0.001
ERI model					
No exposure	41	18.2	184	81.8	
High ER ratio + high overcommitment	93	58.1	67	41.9	<0.001
Combined DCS + ERI models					
No exposure	110	34.0	213	66.0	
Exposure	50	80.6	12	19.4	<0.001

Note: DCS  =  demand-control-support; ERI  =  effort-reward imbalance; ER  =  effort-reward

Violence at the workplace was mentioned by 80.5% of the participants (Table 2), corresponding to: verbal abuse (61.8%), harassment (53.8%), be the target of jokes and discrimination (48.6%), threats of aggression (35.1%), assault at work or on the way to work (29.3%), sexual harassment (12.2%), physical aggression (9.3%) and sexual violence (1.0%).

The results of Cronbach's alpha for the DCSQ dimensions were: demand, 0.73; control: 0.56; and social support, 0.84. High job strain was the most prevalent quadrant in the DCS model (44.9%). Social support was reported to be high by 68.1% of the participants.

The Cronbach's alpha for the ERI questionnaire dimensions was: effort, 0.79; reward, 0.86; and overcommitment, 0.85. The ERI condition was reported by 37.9% of the participants. High overcommitment to work was reported by 64.2% of the participants (Table 2).

Most of the participants (91%) reported having sought medical care in the previous year. The main health problems motivating those medical visits were: emotional disturbances (48.3%) and back pain (35.6%). The average BMI of the participants was 28.84 kg/m^2^ (±5.58 kg/m^2^) representing a tendency to overweight (Table 2).

The individual features selected for multiple modeling were: sex, self-reported skin color, education, level of tobacco use, level of alcohol intake and physical activity and number of morbidities reported in the previous year (Table 1). The selected variables corresponding to occupational factors were: employment status, occupational group, violence at the workplace, DCS model, ERI model, combined DCS and ERI models (Table 2). Although variable participants' age did not showed a p-value equal to or lower than 0.20, it was kept in the final model because it might modify the outcome variable.

All the regression analyses showed that LTSA-MD was associated with the female sex, self-reported white skin color, higher educational levels, high tobacco consumption, high alcohol intake, two or more comorbidities reported in the previous year, exposure to violence at work (Table 3).

**Table 3:** Multiple logistic regression analyses and factors associated with long-term sickness absence due to mental disorders^a^, São Paulo, Brazil, 2011 (N = 385).

	Regression A OR (95%CI)	Regression B OR (95%CI)	Regression C OR (95%CI)	Regression D OR (95%CI)
**Sex**				
Male	1.0 (Ref)	1.0 (Ref)	1.0 (Ref)	1.0 (Ref)
Female	2.22 (1.27–3.87)**	2.09 (1.21–3.62)**	2.07 (1.18–3.64)*	2.30 (1.32–4.01)**
**Self-reported skin color**				
White	1.0 (Ref)	1.0 (Ref)	1.0 (Ref)	1.0 (Ref)
Other	0.50 (0.29–0.84)**	0.51 (0.30–0.86)*	0.49 (0.29–0.84)**	0.49 (0.29–0.83)**
**Education (formal schooling in years)**				
<11	1.0 (Ref)	1.0 (Ref)	1.0 (Ref)	1.0 (Ref)
> = 11	2.61 (1.45–4.70)**	2.41 (1.34–4.32)**	2.51 (1.39–4.56)**	2.43 (1.35–4.37)**
**Tobacco use**				
None	1.0 (Ref)	1.0 (Ref)	1.0 (Ref)	1.0 (Ref)
Low	0.66 (0.29–1.51)	0.65 (0.28–1.50)	0.70 (0.30–1.62)	0.66 (0.29–1.49)
High	8.85 (2.44–32.10)***	7.16 (2.01–25.48)**	8.66 (2.33–32.11)**	8.08 (2.24–29.19)**
**Alcohol intake**				
None	1.0 (Ref)	1.0 (Ref)	1.0 (Ref)	1.0 (Ref)
Low	0.29 (0.12–0.69)**	0.36 (0.16–0.83)*	0.31 (0.13–0.74)**	0.30 (0.13–0.69)**
High	10.04 (1.18–85.63)*	11.05 (1.42–85.90)*	9.98 (1.16–86.11)*	9.07 (1.05–78.12)*
**Comorbidities**				
0 or 1	1.0 (Ref)	1.0 (Ref)	1.0 (Ref)	1.0 (Ref)
2 or more	5.55 (2.81–10.95)***	4.64 (2.36–9.13)***	4.77 (2.38–9.56)***	5.35 (2.73–10.49)***
**Violence at workplace**				
No	1.0 (Ref)	1.0 (Ref)	1.0 (Ref)	1.0 (Ref)
Yes	4.43 (1.96–9.99)***	3.60 (1.59–8.14)**	3.47 (1.51–7.97)**	4.36 (1.94–9.79)***
**DCS model**				
No exposure	1.0 (Ref)		1.0 (Ref)	
High job strain + Low social support	4.43 (2.33–8.45)***		3.37 (1.71–6.66)***	
**ERI Model**				
No exposure		1.0 (Ref)	1.0 (Ref)	
ERI + high overcommitment		3.04 (1.77–5.24)***	2.15 (1.21–3.84)**	
**Interaction of DCS + ERI models**				
No exposure				1.0 (Ref)
High job strain + low social support + ERI + high overcommitment				5.20 (2.40–11.25)***

Note:^ a^ adjusted for age; * p <0.05; ** p<0.01; *** p<0.001; OR  =  odds ratio; 95% CI = 95% confidence interval; DCS  =  demand-control-support; ERI  =  effort-reward imbalance.

Perceived psychosocial factors at work were associated with LTSA-MD. The analyses showed that the both DCS and ERI models were associated with the outcome. Statistical difference was not found between separate or combined analysis of those models dimensions (Table 3).

## Discussion

The case-control design selected for the present study allowed detecting that individual features and psychosocial work conditions were significantly associated with LTSA-MD compared to other causes of sick leave. In addition, dimensions of occupational stress assessed by the DCS and ERI models were significantly associated with LTSA-MD.

### LTSA-MD and Individual Features

The tendency of women to show greater concern about their health might account for the fact that sex was one of the sociodemographic variables associated with LTSA-MD. This female cultural trait might be reflected in an earlier search for medical help, because women deem reporting psychiatric symptoms more acceptable than men do [29]. In addition, the women's frequent assumption of the “double burden” of juggling work and family is also considered to be a factor strongly associated with sick leave episodes [23].

The association of LTSA-MD with self-reported white skin color and high educational levels might be accounted for by the greater access these groups of individuals have to health information in Brazil. More informed workers might detect more easily situations that pose a threat to their mental health [30], and thus seek appropriate actions that might demand sick leave. Those facts notwithstanding, the relevance of the educational level is controversial. A recent review found that high educational levels were protective against, but also a risk factor for longer sickness absence due to mental disorders [22].

Sick leave due to psychiatric disorders is described in male alcohol drinkers and female smokers [31]. The present study showed that both, high alcohol intake and high tobacco consumption, were associated with LTSA-MD regardless of sex. However, as a function of the study design, we were not able to establish the temporal precedence between these habits and mental distress.

Multiple health complaints [32] and self-rated poor health status [7] are considered risk factors for sick leave, and our results corroborate this hypothesis. Reporting two or more comorbidities in the previous year was associated with LTSA-MD. Self-perceived deterioration of the general state of health probably represents one further psychological stressor that increases the workers' mental fatigue. This situation might eventually predispose workers toward absenteeism.

### LTSA-MD and Psychosocial work conditions

Perception of the work conditions as characterized by low control, high demands, low social support, high effort, low reward and high overcommitment exhibited significant association with LTSA-MD. Altogether, those stressors might denote cognitive overload, resulting from excessive demands at work, the effort associated with multitasking, and lack of perspectives for professional growth.

Previous studies showed that all-cause LTSA is associated with high job strain in middle-aged workers [20]. LTSA was also associated with dimensions assessed by the combination of DCS and ERI models among nursing professionals [17]. A recent study conducted in Canada showed that only ERI was independently associated with sick leave due to mental health problems [19].

Job control at work is a part of the theoretical construction of the two models used herein to evaluate occupational stress. This type of psychosocial factor at work combines task performance (micro aspect) with broader issues related with the rewards, such as salary and career progression (macro aspect) [33]. Low control and/or low reward might make mental suffering and fatigue worse. A recent study conducted in Belgium showed that only low reward at work was independently associated with LTSA-MD, although other dimensions of the DCS and ERI models had also been included in analysis [18].

In the ERI model, effort might behave as an extrinsic (like, e.g., work activities) and also as an intrinsic, or personal component (as, e.g., high overcommitment) liable to affect the workers' mental health. Combination of low effort and high demand is significantly associated with medically certified absence for mental health problems [19].

High job strain is an important factor associated with LTSA [20] and this finding agrees with our results relative to sick leave due to mental disorders. However, the results of the Belstress III study did not find association of LTSA-MD with high job strain or social support at work [19].

Both low social support and excessive personal commitment were associated with LTSA-MD in the present study. Workers might sometimes feel themselves alone, while having to behave in a resolute manner vis-à-vis excessive demands relative to which they have little decision-making power [34]. Thus being, one might hypothesize that cases of this study with LTSA-MD could be triggered by the attempts made by workers to excel in an unstable and or competitive job employment [35]–[36]. Further studies might investigate the effect of subjective assessments of productivity or lack of recognition by peers and superiors on absenteeism due to mental sickness.

Exposure to violence at the workplace stood out as an important factor associated with sickness absence, as also an European study found [37]. Bullying, more particularly, is associated with LTSA-MD [18]. This fact is probably due to deterioration of both the vertical (supervisor-subordinate) and the horizontal (between coworkers) work relationships. Exposure to violence at the workplace and low social support at work were associated with LTSA-MD in the present study.

Combined analyses of different scales assessing psychosocial factors at work have the advantage of decreasing the limitations inherent to each individual model [30]–[31], [38]–[40]. In the present study, the model that exhibited the highest odd ratio values was the one that included interactions among dimensions from the two theoretical models of stress used. However, the results of the comparison of the four regression analyses did not indicate statistically significant differences. To the best of our knowledge, the present study was the first to use the aforementioned model analyses to evaluate psychosocial factors at work associated with long-term mental sick leave in workers applying for social security benefits.

There are not legal threshold limit values available for occupational exposure to psychosocial factors at work in spite of their acknowledged negative consequences on the mental health. Those factors should be discussed by managers and subordinates when planning return-to-work actions, in conjunction with preventive measures. Studies on the relationships between work conditions and recurrence of LTSA-MD are needed [21].

Although self-reported data have been used in the investigation of factors associated with absenteeism and illness [17], studies on long-term sickness absence ought to give the preference to data from official registries [41] or confirmed by a physician [42]. One of the strengths of the present study is that both cases and controls were selected based on medical diagnoses confirmed by a highly qualified social security medical expert.

The questionnaires used to investigate the participants' exposure to psychosocial factors at work exhibited satisfactory internal validity, as shown by the Cronbachs alpha values. However, one might question whether interviewing individuals with poor mental health conditions might have not interfered with the results. To be sure, some of the participants might have judged their work conditions as less favorable than they would have done when they were active at work [31], resulting in overestimation of the perceived unfavorable psychosocial factors at work.

Although the study sample consisted of an urban working population holding formal jobs at a major Brazilian city, the results are similar to the ones of other studies conducted in different parts of the world [23], [31], [38]–[39].

## Conclusions

The present study showed that individual features and psychosocial work conditions were associated with long-term sickness absence due to mental disorders. Combined analysis of stress models showed that the psychosocial factors at work were significantly associated with LTSA-MD. The high job strain condition, low social support, effort-reward imbalance and high overcommitment might represent a threat to the mental health of workers. Resourceful use of this information may contribute to the implementation of preventive actions and strategies to facilitate return to work targeting the populations most susceptible to mental disorders.
